# Post-campaign coverage evaluation of a measles and rubella supplementary immunization activity in five districts in India, 2019–2020

**DOI:** 10.1371/journal.pone.0297385

**Published:** 2024-03-29

**Authors:** Jeromie Wesley Vivian Thangaraj, Christine Prosperi, Muthusamy Santhosh Kumar, Alvira Z. Hasan, V. Saravana Kumar, Amy K. Winter, Avi Kumar Bansal, Sanjay L. Chauhan, Gagandeep Singh Grover, Arun Kumar Jain, Ragini N. Kulkarni, Santanu Kumar Sharma, Biju Soman, Itta K. Chaaithanya, Sanchit Kharwal, Sunil K. Mishra, Neha R. Salvi, Nilanju P. Sarmah, Sandeep Sharma, Adarsh Varghese, R. Sabarinathan, Augustine Duraiswamy, D. Sudha Rani, K. Kanagasabai, Abhishek Lachyan, Poonam Gawali, Mitali Kapoor, Saurabh Kumar Chonker, Lucky Sangal, Sanjay M. Mehendale, Gajanan N. Sapkal, Nivedita Gupta, Kyla Hayford, William J. Moss, Manoj V. Murherkar

**Affiliations:** 1 Indian Council of Medical Research (ICMR)-National Institute of Epidemiology, Epidemiology Chennai, Chennai, India; 2 Department of International Health, International Vaccine Access Center, Johns Hopkins Bloomberg School of Public Health, Baltimore, Maryland, United States of America; 3 Department of Epidemiology, Johns Hopkins Bloomberg School of Public Health, Baltimore, MD, United States of America; 4 ICMR-National JALMA Institute for Leprosy & Other Mycobacterial Diseases, Agra, India; 5 ICMR- National Institute for Research in Reproductive and Child Health, Mumbai, India; 6 Directorate of Health Services, Government of Punjab, Chandigarh, India; 7 ICMR-National Institute of Pathology, New Delhi, India; 8 ICMR-Regional Medical Research Centre, Dibrugarh, India; 9 Sree Chitra Tirunal Institute for Medical Sciences and Technology, Achutha Menon Centre for Health Science Studies, Trivandrum, Kerala, India; 10 Department of Health Research, Model Rural Health Research Unit-Dahanu, Maharashtra, India; 11 Department of Health Research, Model Rural Health Research Unit-Hoshiarpur, Punjab, India; 12 Department of Health Research, Model Rural Health Research Unit-Chabua, Assam, India; 13 Department of Health Research, Model Rural Health Research Unit-Kanpur, Uttar Pradesh, India; 14 World Health Organization, Southeast Asia Region Office, New Delhi, India; 15 PD Hinduja Hospital and Medical Research Centre, Mumbai, India; 16 ICMR-National Institute of Virology, Pune, India; 17 Division of Epidemiology and Communicable Diseases, Indian Council of Medical Research, New Delhi, India; International Institute for Population Sciences, INDIA

## Abstract

**Background:**

In alignment with the Measles and Rubella (MR) Strategic Elimination plan, India conducted a mass measles and rubella vaccination campaign across the country between 2017 and 2020 to provide a dose of MR containing vaccine to all children aged 9 months to 15 years. We estimated campaign vaccination coverage in five districts in India and assessed campaign awareness and factors associated with vaccination during the campaign to better understand reasons for not receiving the dose.

**Methods and findings:**

Community-based cross-sectional serosurveys were conducted in five districts of India among children aged 9 months to 15 years after the vaccination campaign. Campaign coverage was estimated based on home-based immunization record or caregiver recall. Campaign coverage was stratified by child- and household-level risk factors and descriptive analyses were performed to assess reasons for not receiving the campaign dose. Three thousand three hundred and fifty-seven children aged 9 months to 15 years at the time of the campaign were enrolled. Campaign coverage among children aged 9 months to 5 years documented or by recall ranged from 74.2% in Kanpur Nagar District to 90.4% in Dibrugarh District, Assam. Similar coverage was observed for older children. Caregiver awareness of the campaign varied from 88.3% in Hoshiarpur District, Punjab to 97.6% in Dibrugarh District, Assam, although 8% of children whose caregivers were aware of the campaign were not vaccinated during the campaign. Failure to receive the campaign dose was associated with urban settings, low maternal education, and lack of school attendance although the associations varied by district.

**Conclusion:**

Awareness of the MR vaccination campaign was high; however, campaign coverage varied by district and did not reach the elimination target of 95% coverage in any of the districts studied. Areas with lower coverage among younger children must be prioritized by strengthening the routine immunization programme and implementing strategies to identify and reach under-vaccinated children.

## Introduction

Measles and rubella (MR) have been targeted for elimination in all World Health Organization (WHO) regions as they are highly contagious vaccine-preventable diseases that can cause significant morbidity and mortality among children and the developing fetus. More than 50% of measles cases and 80% of rubella cases in the South East Asian region are reported from India [1]. India is one of the 11-member states in South East Asia committed to eliminate measles and rubella by 2023. One of the core components to achieve elimination is to maintain high population immunity with at least 95% vaccination coverage with two doses of measles and rubella containing vaccines through routine and/or supplementary immunization [2].

Measles containing vaccine (MCV1) was introduced in the National Immunization Programme (NIP) in 1985 as a single dose for infants aged 9 months and older [3]. The second dose (MCV2) was introduced in the NIP in 2010 across all states of India through a selective supplementary immunization strategy called “Measles catch up campaign” [3,4]. In alignment with the South-East Asian Measles and Rubella Strategic Elimination Plan 2010–2020, India conducted a mass measles and rubella vaccination campaign across the country between 2017 and 2020. The objective of the campaign was to provide a single dose of measles and rubella containing vaccine to all children aged 9 months to <15 years and to introduce the rubella containing vaccine into the NIP [3]. Following the campaign, India implemented a two dose MR vaccine strategy in the routine immunization programme, with the first dose given between 9–12 months and second between 16–24 months of age [5].

The MR vaccination campaign targeted more than 400 million children and was implemented in phases across India. The vaccination campaign was conducted in schools targeting children aged between 5 and <15 years and was followed by implementation in outreach sites in the community to vaccinate younger children between 9 months and 5 years of age [3]. The campaign in each state was planned for 3 to 4 weeks and was extended by one or two weeks to improve coverage.

The campaign faced many challenges and critical issues during implementation in the first phase in the states and Union Territories of Goa, Karnataka, Lakshadweep, Puducherry and Tamil Nadu [3]. Rumors were spread about the safety of the vaccines, especially on social media, and campaign managers were not adequately prepared to handle such negative messages. Other issues included poor sensitization of the school authorities and private practitioners regarding the usefulness of MR vaccination, absence of intersectoral coordination between education and health departments, and lack of information, education and communication materials customized for teachers and parents [6–13]. However, these issues were adequately dealt with during the planning of subsequent phases of the MR vaccination campaign [3].

Post-coverage evaluation surveys were recommended to document the effectiveness of the MR campaign and to guide the programme on elimination strategies but were only conducted in three states of India [6,7,9,14]. Apart from vaccination coverage estimates, these surveys help to document reasons children were not reached through the campaign to assist in designing and planning future SIAs. We conducted cross-sectional serosurveys before and after the 2017–2019 MR SIA, including four districts with data from both time points and one district with post-SIA data only. All SIAs in these districts were conducted in the later phases of the campaign. Prior publications presented measles and rubella seroprevalence, vaccination coverage, and correlations between routine and campaign coverage [15,16]. In this paper we focus on the data related to the MR campaign from the post-campaign serosurveys, including estimating MR campaign vaccination coverage, identifying factors associated with receipt of the campaign dose and reported reasons for non-receipt of the campaign dose, and explored the effectiveness of the communication strategy in promoting campaign awareness.

## Methods

### Survey setting

Community-based cross-sectional serosurveys were conducted in five districts of India (Dibrugarh District, Assam; Hoshiarpur District, Punjab; Palghar District, Maharashtra; Kanpur Nagar District, Uttar Pradesh (UP); and Thiruvananthapuram District, Kerala) after the MR vaccination campaign conducted from 2017 to 2020. The surveys were conducted from 2018 to 2020 across two age groups (9 months to younger than 5 years and 5 years to younger than 15 years). The goal of each survey was to estimate measles and rubella seroprevalence and to evaluate the impact of the vaccination campaign [15]. This analysis is restricted to children enrolled in the surveys conducted after the MR campaign in the five survey districts. The surveys were conducted between three to 16 months after the MR campaign (Table 1). These districts varied in characteristics that impact vaccination coverage, such as rural and urban differences, health care quality, adult literacy, and access to private healthcare.

**Table 1:** Individual and household-level characteristics among children enrolled in the post-SIA survey.

	Thiruvananthapuram District, Kerala	Kanpur Nagar District, Uttar Pradesh	Palghar District, Maharashtra	Hoshiarpur District, Punjab	Dibrugarh District, Assam
	n (%)	n (%)	n (%)	n (%)	n (%)
**Approximate time between SIA and survey** ^a^	10–16 m	3–6 m	3–5 m	13–16 m	12–14 m
Age group					
9 months to < 5 years	340	341	336	346	312
5 to < 15 years	338	350	321	348	325
Female	330 (48.7)	320 (46.3)	341 (51.9)	340 (49.0)	312 (49.0)
Maternal education level					
Graduate or above	240 (35.5)	128 (18.7)	78 (12.0)	115 (16.7)	41 (6.6)
Middle to higher secondary	395 (58.3)	290 (42.4)	326 (50.2)	463 (67.3)	296 (47.6)
Primary	35 (5.2)	115 (16.8)	77 (11.8)	60 (8.7)	235 (37.8)
Illiterate	7 (1.0)	151 (22.1)	169 (26.0)	49 (7.1)	50 (8.0)
Head of household occupation					
Professional, technician, clerks	138 (20.4)	124 (17.9)	77 (11.7)	61 (9.0)	89 (14.0)
Service & sales workers, agriculture, craft trade	435 (64.2)	506 (73.2)	545 (83.1)	551 (81.4)	531 (83.8)
Unemployed	105 (15.5)	61 (8.8)	34 (5.2)	65 (9.6)	14 (2.2)
Household materials					
Permanent	446 (65.8)	393 (56.9)	295 (44.9)	620 (90.2)	98 (15.5)
Semi-permanent	171 (25.2)	231 (33.4)	248 (37.7)	40 (5.8)	94 (14.8)
Non-permanent	61 (9.0)	67 (9.7)	114 (17.4)	27 (3.9)	442 (69.7)
Setting					
Rural	348 (51.3)	246 (35.6)	319 (48.6)	452 (65.4)	496 (77.9)
Urban Slum	114 (16.8)	255 (36.9)	163 (24.8)	24 (3.5)	65 (10.2)
Urban Non-slum	216 (31.9)	190 (27.5)	175 (26.6)	215 (31.1)	76 (11.9)
Religion					
Hindu	514 (75.8)	623 (90.2)	594 (90.4)	469 (68.3)	597 (94.2)
Muslim / Christian	164 (24.2)	68 (9.8)	44 (6.7)	11 (1.6)	36 (5.7)
Sikhs / Buddhist / Jain	0 (0.0)	0 (0.0)	19 (2.9)	207 (30.1)	1 (0.2)
Caste					
General / Other Backward Class	529 (78.7)	480 (69.5)	369 (56.9)	481 (70.0)	521 (84.3)
Scheduled Caste / Tribe	143 (21.3)	211 (30.5)	279 (43.1)	206 (30.0)	97 (15.7)
Campaign card availability^b^					
9 months to < 5 years	34 (10.3)	161 (47.6)	255 (76.1)	211 (61.7)	201 (65.5)
5 to < 15 years	24 (7.6)	174 (50.0)	241 (76.5)	206 (60.6)	217 (70.7)
Campaign card not available	620 (91.4)	356 (51.5)	159 (24.3)	275 (39.7)	218 (34.3)
Campaign card lost^c^	255 (37.6)	154 (22.3)	107 (16.3)	156 (22.5)	151 (23.7)
Did not receive campaign card^c^	334 (49.3)	197 (28.5)	47 (7.2)	109 (15.8)	45 (7.1)
Unknown reason for missing campaign card^c^	31 (4.6)	5 (0.7)	5 (0.8)	10 (1.5)	22 (3.5)
Aware of the campaign	630 (92.9)	616 (89.1)	630 (96.0)	613 (88.3)	622 (97.6)

^a.^ Approximate time between campaign and survey range from 3–6 months in Palghar District and Kanpur Nagar District to over 10 months in the other districts (Thiruvananthapuram, 10–16 m; Hoshiarpur, 13–16 m; Dibrugarh, 12–14 m). Duration is based on approximate district-level campaign start date (minimum) and stop date (maximum) and the date the serosurvey was started.

^b.^ Card available and shown to interviewer.

^c.^ Percentages calculated out of all children enrolled.

### Survey methods

A three-stage cluster survey design was implemented. Detailed methods were reported elsewhere and followed recommendations from the WHO Vaccination Survey Manual [17]. Briefly, for each survey, villages or wards were selected from each district based on the 2011 census, one census enumeration block was randomly selected from each village, and all eligible individuals in a cluster were enumerated and then randomly selected for the study. In each cluster, 13 individuals were selected per age group. Sample size was estimated for the overall serosurvey conducted in these districts, assuming rubella seroprevalence of 50% among both the younger and older age groups, with an absolute precision of 10%, a design effect of 2, and a 95% confidence level.

Prior to enrollment, informed written consent was obtained from all parents or care givers of the child. After obtaining written consent, survey staff collected information on socio-demographic characteristics and vaccination history from parents or caregivers of the selected child. Routine measles vaccination status was only collected from children 9 months to <5 years and was assessed based on caregiver’s recall or vaccination card. Receipt of MR campaign dose was collected from all children aged 9 months to <15 years and assessed based on caregiver’s recall or campaign card. In some cases, MR campaign doses were recorded in other documents such as the routine vaccination card owing to reported shortages of campaign card supplies. Field teams recorded such doses as recalled. A venous blood sample was collected to test for IgG antibodies to measles and rubella viruses.

The surveys were led by the Indian Council of Medical Research (ICMR) National Institute of Epidemiology (NIE), Chennai, and locally implemented by researchers at the Model Rural Health Research Unit (MRHRU) or another research institute in each district. The Institutional Ethics Committees of Indian Council of Medical Research National Institute of Epidemiology, Chennai, India; Johns Hopkins Bloomberg School of Public Health, Baltimore, USA, and the individual study sites approved the protocol. The study team had access to identifiable information during data collection but deidentified datasets were used in analyses.

### Analysis

Descriptive analyses of child and household-level characteristics of all enrolled children aged 9 months to <15 years at the time of the campaign were conducted. Campaign coverage was estimated based on home-based (vaccine card) record documentation only, recall only, and using both sources of information. MR campaign coverage was stratified by child- and household-level risk factors, including school attendance. Coverage estimates with 95% logit confidence intervals accounted for sampling weights, including the selection of the village or ward from the district, selection of the CEB, segmentation within the selected CEB if applicable, and the probability of selecting the child from all enumerated children in the cluster. Cluster-specific unweighted coverage estimates were plotted to explore differences by cluster [17]. Unweighted descriptive analyses were also performed for other campaign related data, including sources of information, location of receipt, and reasons for not receiving the campaign dose.

District-specific univariable regression analyses accounting for the survey design were run to explore associations with whether a child received the campaign dose. The following variables were considered in the analysis: child’s sex, head of household occupation, type of schooling, maternal education, setting, religion, caste, and age at the campaign. Variables with a p-value < 0.25 from the district-specific univariable analyses were included in the district-specific multivariable analyses. Removing additional variables from the district-specific multivariable models had minimal impact on the model fit as evaluated using AIC; therefore, no further adjustments were made to the models.

The Integrated Child Development Scheme as a part of early childhood care and education provides services related to health including NIP vaccination, nutrition, and play and early learning for children from birth to six years of life through Anganwadi centres. Play school in Anganwadi centres begins at three years of age [18]. Unless otherwise specified by the caregiver, children younger than three years of age were treated as too young to attend school and any child older than this age who was not in school was treated as such in analyses.

MR campaign vaccination status was missing for 3–17 (0.4% - 2.4%) campaign responses, ranging by district. Those missing data were treated as not having received the dose. All analyses were conducted using SAS version 9.4 and R version 4.2.1.

## Results

### Characteristics of the survey sample

Three thousand three hundred and fifty-seven children aged 9 months to <15 years at the time of the campaign were enrolled in surveys across five districts in India after the MR campaign (Table 1 and S1 Fig). The sex distribution of enrolled children was similar across all districts (range for females: 46.3% in Kanpur Nagar to 51.9% in Palghar). The maternal education level of participants was highest in Thiruvananthapuram and lowest in Palghar and Kanpur Nagar Districts, but in all districts most mothers received middle to higher secondary education. Survey clusters in Hoshiarpur and Dibrugarh were primarily located in rural settings (65.4% and 77.9% of participants, respectively) while in Kanpur Nagar children were primarily located in urban settings (36.9% in urban slum and 27.5% in urban non-slum). In the remaining districts, the clusters were equally distributed across rural and urban (slum/non-slum) settings. Across districts most participants were of Hindu religion and belonged to general or other backward class caste.

Availability of campaign cards varied by district in both age groups, ranging from 76.1% in Palghar to 10% or less in Thiruvananthapuram District, Kerala. The common reasons for not having a campaign card included loss of card between the SIA and survey (range: 16.3% in Palghar to 37.6% in Thiruvananthapuram) and not receiving a card at the time of the campaign, which primarily occurred in Thiruvananthapuram (49.3%) and Kanpur Nagar (28.5%). In Thiruvananthapuram District, the campaign card availability was low due to operational reasons such as limited availability of cards.

### MR campaign vaccination coverage and awareness

**Fig 1:**
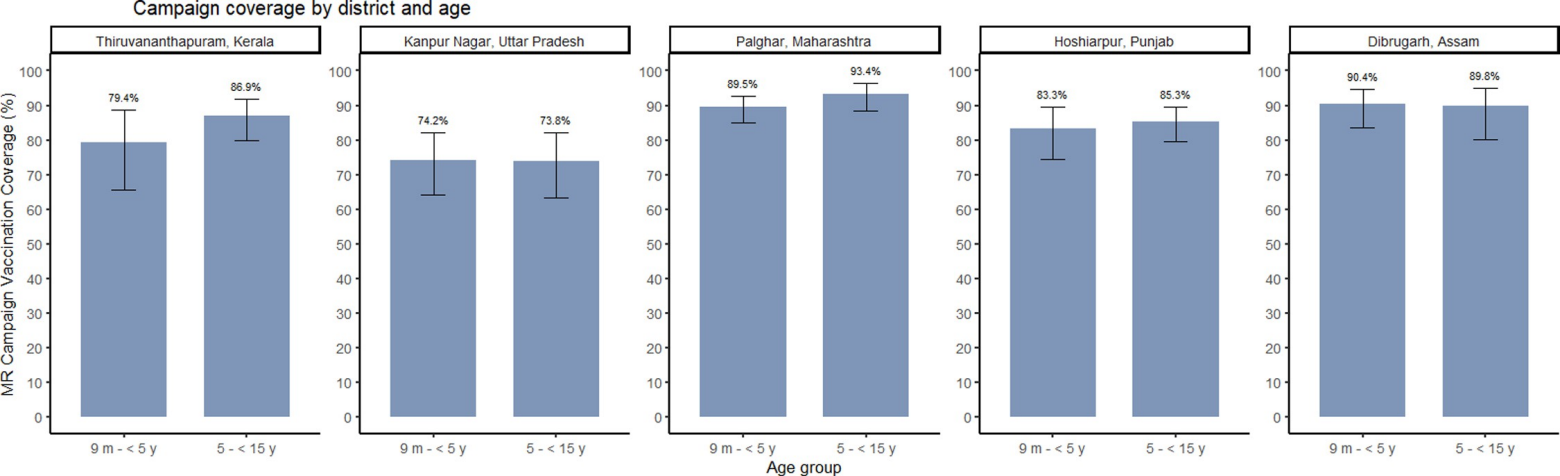
Measles-rubella campaign vaccine coverage by district and age group. Survey weighted coverage estimates. Bars represent 95% confidence intervals.

Campaign coverage among children aged 9 months to <5 years either documented or by recall ranged from 74.2% (95% CI: 64.1, 82.2) in Kanpur Nagar District, Uttar Pradesh to 90.4% (95% CI: 83.7, 94.6) in Dibrugarh District, Assam (Fig 1 and S1 Table and S2 Fig). The campaign coverage among children aged 5 to <15 years either documented or by recall ranged from 73.8% (95% CI: 63.2, 82.2) in Kanpur Nagar District, UP to 93.4% (95% CI: 88.6, 96.3) in Palghar District, Maharashtra. No difference in campaign coverage by age group was observed except in Thiruvananthapuram District, Kerala where coverage was higher among older children. Most campaign coverage was documented on a vaccination card except in Thiruvananthapuram District, Kerala where documented campaign doses accounted for only 8.7% (95% CI: 5.5, 13.5) and 5.2% (95% CI: 2.7, 9.6) of the campaign coverage estimates in younger and older age groups, respectively. Coverage among zero-dose children ranged from 45.7% (95% CI: 21.8, 71.8) in Kanpur Nagar District, UP to 93.7% (95% CI: 73.1, 98.8) in Dibrugarh District, Assam (S1 Table). Cluster-level coverage varied substantially, particularly in Thiruvananthapuram District, Kerala and Kanpur Nagar District, UP (S3 Fig).

Awareness of the campaign among caregivers varied from 88.3% in Hoshiarpur District, Punjab to 97.6% in Dibrugarh District, Assam (Table 1). Most caregivers who were aware of the campaign learned about the campaign from a health worker (range: 29.3% in Hoshiarpur to 62.5% in Dibrugarh) or from a school or a community member (e.g., leader, religious head, or neighbor) (30.8% in Dibrugarh to 55.9% in Hoshiarpur) (S2 Table). In Thiruvananthapuram, Kanpur Nagar and Dibrugarh Districts, over 50% of caregivers whose child did not receive the campaign dose reported being aware of the campaign (S3 Table).

### Campaign coverage by child and household characteristics

No significant differences in campaign coverage by sex were observed except in Dibrugarh District, Assam with lower coverage among boys relative to girls (Tables 2 and S4 and S5). In the younger age group in Kanpur Nagar District, Uttar Pradesh and Thiruvananthapuram, Kerala, girls had lower coverage than boys (69.3% vs 79.0% in Kanpur Nagar District; 75.9% vs. 82.6% in Thiruvananthapuram District) (S6 Table). Campaign coverage differed by maternal education in some but not all sites. In Hoshiarpur and Thiruvananthapuram Districts, vaccination coverage was lowest among children whose mother was illiterate or had primary level of education. In Palghar District, Maharashtra differences by maternal education were also observed but all education categories had coverage >85%, with the lowest coverage in the highest educated group. Lower coverage was observed among children whose parents were unemployed in Thiruvananthapuram, Kanpur Nagar, and Dibrugarh Districts. Campaign coverage was highest among children living in rural settings relative to urban settings, and most pronounced in Kanpur Nagar (rural: 85.6%, urban slum: 58.5%) and Dibrugarh (rural: 95.6%, urban slum: 84.9%, urban non-slum: 69.3%) District. No significant difference by setting was observed in Hoshiarpur or Thiruvananthapuram Districts.

**Table 2:** Campaign coverage by individual and household characteristics, children aged 9 months to younger than 15 years.

		Thiruvananthapuram, Kerala % (95% CI)	Kanpur Nagar, Uttar Pradesh % (95% CI)	Palghar, Maharashtra % (95% CI)	Hoshiarpur, Punjab % (95% CI)	Dibrugarh, Assam % (95% CI)
Sex	Male	85.5 (77.1, 91.2)	74.8 (64.9, 82.7)	91.2 (85.2, 94.9)	85.5 (79.4, 90.1)	**88.3 (78.3, 94.0)**
	Female	83.2 (72.2, 90.5)	72.9 (62.1, 81.5)	93.1 (88.0, 96.1)	83.8 (77.0, 88.8)	**92.0 (85.6, 95.7)**
Maternal education	Graduate and above	**82.1 (61.6, 92.9)**	81.1 (66.6, 90.2)	**85.2 (61.0, 95.5)**	**86.0 (75.8, 92.3)**	92.3 (77.7, 97.6)
	Middle to higher secondary	**88.0 (81.1, 92.7)**	77.6 (68.8, 84.4)	**95.5 (92.2, 97.4)**	**85.8 (80.1, 90.0)**	89.6 (81.0, 94.6)
	Primary	**58.4 (28.4, 83.2)**	78.0 (60.1, 89.3)	**90.2 (76.0, 96.4)**	**91.0 (77.1, 96.8)**	92.8 (81.8, 97.4)
	Illiterate	**83.7 (62.0, 94.2)**	63.0 (48.6, 75.4)	**89.7 (82.5, 94.1)**	**68.6 (43.0, 86.4)**	83.9 (48.7, 96.6)
Head of household occupation	Professional, technician, clerks	**80.1 (49.2, 94.4)**	76.9 (65.2, 85.6)	93.1 (83.0, 97.4)	90.7 (74.3, 97.1)	**86.3 (73.7, 93.4)**
	Service & sales workers, agriculture, craft trade	**88.5 (80.9, 93.3)**	75.1 (63.2, 84.1)	91.6 (87.5, 94.5)	83.3 (77.4, 88.0)	**91.9 (85.6, 95.6)**
	Unemployed	**74.2 (60.7, 84.3)**	54.5 (36.6, 71.4)	97.5 (89.3, 99.5)	87.7 (71.8, 95.3)	**59.7 (16.9, 91.5)**
Setting	Rural	88.1 (77.0, 94.3)	**85.6 (75.6, 91.9)**	**94.9 (89.2, 97.7)**	83.5 (75.4, 89.3)	**95.6 (91.8, 97.7)**
	Urban Slum	86.8 (79.5, 91.8)	**58.5 (43.6, 72.1)**	**88.4 (83.6, 92.0)**	89.1 (89.1, 89.1)	**84.9 (66.0, 94.3)**
	Urban Non-slum	75.9 (52.7, 89.9)	**76.3 (64.7, 85.0)**	**92.1 (88.5, 94.7)**	86.4 (80.2, 90.8)	**69.3 (63.8, 74.4)**
Religion	Hindu	86.4 (77.3, 92.2)	76.0 (65.1, 84.3)	**93.5 (90.2, 95.7)**	**86.3 (81.3, 90.1)**	90.1 (82.3, 94.7)
	Muslim / Christian	79.1 (64.8, 88.7)	57.5 (34.5, 77.6)	**73.5 (42.3, 91.3)**	**40.1 (10.2, 79.9)**	88.3 (52.7, 98.1)
	Sikhs / Buddhist / Jain	N/A	N/A	**86.5 (60.5, 96.4)**	**84.0 (75.4, 90.0)**	N/A^a^
Caste	General / Other Backward Class	84.4 (74.5, 90.8)	76.7 (67.7, 83.8)	90.9 (86.3, 94.1)	84.4 (78.2, 89.1)	90.0 (81.5, 94.9)
	Scheduled Caste / Tribe	83.8 (64.9, 93.5)	68.5 (51.9, 81.4)	94.2 (88.8, 97.1)	85.4 (78.5, 90.3)	89.5 (74.0, 96.3)
School attendance	Public school	**87.2 (79.1, 92.5)**	77.7 (66.0, 86.3)	**93.7 (87.5, 97.0)**	**88.3 (78.4, 94.0)**	**93.2 (86.3, 96.8)**
	Private school	**83.0 (66.0, 92.4)**	75.9 (67.5, 82.7)	**97.4 (91.3, 99.3)**	**87.1 (81.2, 91.4)**	**86.6 (73.1, 93.9)**
	Too young to attend school^b^	**64.5 (45.5, 79.8)**	75.6 (64.8, 83.9)	**88.8 (84.0, 92.3)**	**55.8 (31.4, 77.7)**	**82.0 (68.3, 90.6)**
	Does not attend school^b^	**100.0 (100.0, 100.0)**	36.2 (13.1, 68.1)	**82.2 (70.3, 90.0)**	**14.9 (2.83, 51.2)**	**70.4 (34.2, 91.6)**

Percent of children who received the campaign dose within each site and category (row percentages, survey weighted estimates). Bold indicates factor is significantly associated with campaign coverage (p-value from multivariable logistic regression model < 0.05). See S4 and S5 Tables for the regression output.

^a.^ Due to small numbers in the category (N = 1) no coverage estimate was calculated and this child was excluded from regression analyses.

^b.^ Due to confusion during the conduct of the survey between ‘does not attend’ and ‘too young’ options, 3 years of age was used to distinguish ‘too young’ from ‘does not attend’ for the analysis: Children younger than 3 years at the time of the campaign marked as ‘does not attend’ were considered ‘too young’, and children 3 years or older at the time of the campaign marked as ‘too young’ were considered ‘does not attend’.

Campaign coverage differed by school attendance in most districts, with those attending school having the highest levels of coverage compared to those too young to attend school or not attending school (Table 2). Campaign coverage differed by type of schooling in Dibrugarh District, Assam with higher coverage observed among children attending public school compared to private school, although coverage was above 85% in both types of schools.

Among children older than three years of age who received the campaign dose, over 70% received the campaign dose at school (S7 Table). In Kerala and Palghar Districts children attending private schools were more likely to have received the campaign dose in a school-setting compared to public school-going children; however, the reverse was observed in Hoshiarpur District (S8 Table). Children too young to attend school or not attending school tended to be vaccinated at a government health facility (Thiruvananthapuram District) or outreach site (Kanpur Nagar, Palghar, and Hoshiarpur Districts). In Dibrugarh District, Assam most children who received the vaccine did so at a school whether or not they attended school, although the school may have served as an outreach location for those not attending school (S8 Table).

### Reasons children were missed during the campaign

Among children who did not receive a campaign dose, the primary reason reported for not receiving a campaign dose was lack of knowledge about the campaign except in Palghar District, Maharashtra where living outside the state during the SIA was the most common reason (39.5%) (Table 3). Other common reasons included lack of faith in vaccines (23.6% in Hoshiarpur District, Punjab) and sickness at the time of the campaign (20% in Dibrugarh District, Assam).

**Table 3:** Reasons for not receiving the campaign vaccine for children aged 9 months to younger than 15 years.

Site	N	Lack of knowledge of campaign (when or where occurring)	Lack of faith in vaccines or vaccine campaigns	Lack of time or other issue (too busy; family problem)	Fear of pain and other side effects from injection/fear of vaccines	Child sick	Did not live in state during MR campaign	Heard vaccine may harm child	Don’t remember	Other
Thiruvananthapuram District, Kerala	97	50 (51.5)	4 (4.1)	1 (1.0)	5 (5.2)	6 (6.2)	5 (5.2)	4 (4.1)	9 (9.3)	13 (13.4)
Kanpur Nagar District, Uttar Pradesh	181	70 (38.7)	13 (7.2)	12 (6.6)	28 (15.5)	17 (9.4)	25 (13.8)	10 (5.5)	1 (0.6)	5 (2.8)
Palghar District, Maharashtra	38	9 (23.7)	0 (0.0)	3 (7.9)	1 (2.6)	1 (2.6)	15 (39.5)	3 (7.9)	3 (7.9)	3 (7.9)
Hoshiarpur District, Punjab	106	48 (45.3)	25 (23.6)	0 (0.0)	1 (0.9)	8 (7.5)	8 (7.5)	12 (11.3)	0 (0.0)	4 (3.8)
Dibrugarh District, Assam	60	26 (43.3)	0 (0.0)	2 (3.3)	2 (3.3)	12 (20.0)	2 (3.3)	0 (0)	8 (13.3)	8 (13.3)

Reflects the first reason reported if more than one was reported. “Heard vaccine may harm child” from either social or other media sources, religious leader, or other community member. “Other” includes service delivery issue, receipt of vaccine from private provider, or school opted out of the campaign (less than 3% of children in all site analysis).

When restricting to children who did not receive an SIA dose but their caregivers indicated they were aware of the campaign, common reasons reported for not receiving an SIA dose were the child being sick at the time of the campaign (15.7%), living out of state at the time of the campaign (14.5%), and fear of pain or side effects of the vaccine (14.1%) (S9 Table).

## Discussion

Nearly 4 out of 5 eligible children received the MR vaccine dose during the campaign in all study districts except Kanpur Nagar, Uttar Pradesh where the vaccination coverage was approximately 75%. Campaign awareness was high in all districts but 8% of caregivers who were aware did not have their child receive the campaign dose. We observed differences in campaign dose receipt by rural/urban setting, maternal education, school attendance, and type of schooling, although the associations varied by district.

These coverage estimates are consistent with MR seroprevalence data from the same survey, in which we observed the lowest measles seroprevalence following the MR campaign in Kanpur Nagar District and no change in seroprevalence when compared to the serosurvey conducted in the district prior to the campaign [15]. MR vaccination campaign coverage estimated by other studies ranged between 90.5% in Tamil Nadu to 68.6% in Imphal [6,7,9,14]. Most surveys were conducted at sub-district levels immediately after the campaign. Evaluation coverage surveys conducted after the MR campaign in the neighboring countries of Bangladesh in 2014 and Nepal in 2016 estimated MR coverage around 90% and 85% respectively [19,20].

Vaccination coverage was higher among children attending school compared to those too young to attend school or not attending school. This is likely due to the greater focus given to the school vaccination strategy, availability of most children in school at the time of the campaign, and cooperation from the private and public-school authorities. Monitoring and reviews conducted during the initial phases of the campaign indicated gaps in the pre-campaign information, education and communication materials provided to the teachers, parents and local leaders/influencers [10,21]. Critical issues identified in the initial phase were rectified in the later phases of the campaign planning and implementation. In all study districts, the MR campaign was conducted in the later phases and hence may have benefitted from the lessons learnt from the initial campaign districts. The strategy used in schools was improved by increasing the duration of the activity, such as scheduling SIA sessions both in the morning and afternoon. Lists of schools and students were prepared by the education department to ensure full coverage. The principal and one nodal person were made responsible for coordination with the health department and a class teacher and 2–3 students from each class were assigned to support the MR campaign [3]. Schools and school teachers also played a crucial role in motivating parents to vaccinate their children during the MR campaign, as demonstrated by a qualitative study conducted in Tamil Nadu where vaccine acceptance was higher in schools because parents trusted the health information provided by teachers [11]. Although differences in coverage by school attendance was observed in all districts, in Palghar District, Maharashtra the campaign dose receipt was greater than 88% even among those too young to attend school or not attending school.

Earlier studies indicated that childhood vaccination uptake is better among those with mothers who had higher educational qualifications [22]. In our study, receipt of the MR vaccination campaign dose was associated with mother’s education status and head of the household occupation. Coverage tended to be lower among illiterate and unemployed parents, although the associations and direction varied by district. In Palghar District of Maharashtra, coverage was slightly lower among the highest educated mothers, although 85% or higher for all categories of maternal education. This finding is similar to that from a MR campaign evaluation study conducted in Tamil Nadu that found vaccination coverage was lower among more educated mothers primarily due to the adverse social media messages that were circulating during the initial phases of the campaign [7].

MR campaign coverage was higher in rural areas than urban areas in all study districts except Hoshiarpur District of Punjab and Thiruvananthapuram District of Kerala. Especially in Kanpur Nagar, coverage was lower among urban-slum dwellers. In India several surveys have highlighted higher vaccination rates in rural than urban areas. This might be due to challenges such as migrant populations, inadequate microplanning, and lack of human resources for health and infrastructure in some urban areas [23].

Campaign awareness was high (more than 88%) in all study districts. In Dibrugarh, Assam and Palghar, Maharashtra, nearly 97% of caregivers knew about the campaign. Most mothers and caregivers reported that they knew about the campaign either from a health care worker or school or community/neighborhood member, with few people reporting social media as a source of information about the campaign. In the later phases of the campaign, renewed emphasis was placed on effective communication and public engagement with parents and schools, health professionals, community leaders and the media, to gain their trust, leveraging key influencers to explain the benefits of immunization and address community concerns [3,10,13]. Operational guidelines mandated that healthcare workers visit eligible children’s households and provide interpersonal communication (IPC) through an invitation card at least 3 days before the activity [3]. Most schools issued letters to parents about the campaign and sought permission of parents for their child’s participation in the campaign. The revised guidelines provided the implementing states adequate time to thoroughly prepare for planning and communication activities. Systematic planning and implementation of the IPC activities gave visibility to the campaign and might have been the reason for the high level of awareness regarding the campaign in the community.

The major reason for non-receipt of the MR vaccine campaign dose in the study districts was lack of awareness regarding when and where the vaccination campaign was happening, except in Palghar where the most common reason was that the child was living outside the state. Other reasons such as lack of faith in the vaccines and fear of side effects due to vaccination were also reported. The reasons for non-receipt of MR dose were similar to reasons reported in other studies [9,14,20]. Almost 3–18% of the population across the study districts did not receive the campaign vaccine dose even after they were made aware of the MR campaign vaccination. The primary reasons were that the child was sick, fear of pain and side effects due to vaccines, and the child not available during the campaign. Campaigns are short-term activities and it is possible to miss out on some children either because they are out of town or if they are sick. Repeated visits to households during mop up activities and guidance on alleviating fear of vaccines using an influencer in the community through follow-up activities are necessary to vaccinate the missed children during campaign.

The initial phase of the campaign was disrupted due to anti-vaccination propaganda messages in social media and people believed in fake rumors flooding social media. However, such negative messages as a deterrent for being vaccinated in the campaign was not observed in this study. This reflects the effective communication implemented in the campaign during the preparedness stage in each state to handle negative messages about the safety of vaccines.

Our study has certain limitations. The survey was carried out 3–6 months after the campaign in Palghar, Maharashtra and Kanpur Nagar, Uttar Pradesh and up to 16 months in all the other districts. Recall about awareness, source of information, location of receipt and reasons for not receiving the campaign vaccine may have been impacted by longer durations between the implementation of the campaign and this study in some districts. Campaign card availability was low in Thiruvananthapuram, Kerala and Kanpur Nagar, Uttar Pradesh, hence, coverage estimates were mostly based on recall in these districts. Since older children were provided campaign dose in schools, caregivers might not have remembered the dose. However, in younger children there is a possibility that they might have mistaken the campaign dose with routine vaccination. To better know the reasons for not getting the dose, the data collectors prompted the caregivers for responses. The data collectors had to match the response with the given options in the data collection tool and matching was dependent on the data collectors understanding of the options and interpretation of the caregiver’s response. Misattribution might have occurred as a consequence. Despite these limitations, there were many strengths, such as following recommendations from the WHO cluster survey reference manual including random selection of survey participants, weighted coverage analysis, sampling a fixed numbers of households per cluster rather than fixed number of completed responses, as well as the additional serological data collected to assess immunity gaps.

### Conclusions and recommendations

Although campaign awareness was high, the MR campaign did not reach the elimination target of 95% MR vaccination coverage in all the study districts. Younger children who missed the campaign dose would have an opportunity to receive the MR vaccine through the routine immunization programme but older children would not. Hence, areas with lower coverage among younger children must be prioritized by strengthening the routine immunization programme and implementing strategies to identify and reach under-vaccinated children. School based vaccination strategies achieved higher vaccination than the outreach community-based sessions. In future SIAs, the communication plan to mobilize children to the outreach sessions needs to be strengthened.

## Supporting information

S1 Checklist STROBE statement—checklist of items that should be included in reports of observational studies. (DOCX)

S1 Fig Flowchart describing the enrolment of participants in the post-SIA serosurveys by district. (DOCX)

S2 Fig Coverage of measles-rubella campaign vaccine by district and age group, stratified by source of information on vaccination status. Survey weighted coverage estimates.
(TIF)

S3 Fig Unweighted cluster-level measles-rubella campaign vaccination coverage by cluster setting. Unweighted coverage estimates.
(TIF)

S1 Table Measles-rubella campaign vaccine coverage. (DOCX)

S2 Table Primary source of information about measles-rubella campaign. (DOCX)

S3 Table Campaign coverage by awareness of campaign. (DOCX)

S4 Table Factors associated with receipt of the campaign dose, district-specific univariable logistic regression models. (DOCX)

S5 Table Factors associated with receipt of the campaign dose, district-specific multivariable logistic regression models. (DOCX)

S6 Table Campaign coverage by sex and age group. (DOCX)

S7 Table Location of measles-rubella campaign receipt, by child’s age at the time of the campaign. (DOCX)

S8 Table Location of measles-rubella campaign receipt, by school attendance. (DOCX)

S9 Table Reasons for not receiving the campaign vaccine, children aged 9 months to less than 15 years, restricted to children aware of the campaign. (PDF)
