# Supplementary material for: Post-campaign coverage evaluation of a measles and rubella supplementary immunization activity in five districts in India, 2019–2020
Source: PLoS One. 2024 Mar 29;19(3):e0297385. doi: 10.1371/journal.pone.0297385 (PMC10980234; doi:10.1371/journal.pone.0297385)
Supplement: S1 Fig — (DOCX) [file pone.0297385.s002.docx]

**Supplementary Figure 1-A: Flowchart describing the enrolment of participants in the post-SIA serosurveys by district**

**Hoshiarpur, Punjab**

**Household in the study clusters**

**Enumerated**

**Not Enumerated**

Households

**Post-SIA survey**

3002 Households

2648 (88.2%) households

354 (11.8%) Households

278 (9.3%) Locked

45 (1.5%) Refused

31 (1.0%) Other

658

1574

635 (96.5%)

1490 (94.7%)

390

390

Individuals

Enumerated

Available

Randomly Selected

346 (88.7%)

347 (88.9%)

Enrolled & analysed

**Enumerated:** Visited all households in the cluster and collect identification details (name, date of birth/age, gender) **Available:** Children are available for the next three day**s Randomly selected:** Automated selection of children from enumeration data using android application. **Enrolled and analysed:** Data collected after obtaining consent/assent

9 m - <5 years children

5 - <15 years children

**Supplementary Figure 1-B: Flowchart describing the enrolment of participants in the post-SIA serosurveys by district**

**Dibrugarh, Assam**

**Post-SIA survey**

1914 (78.8%) households

514 (21.2%) Households

427 (17.6%) Locked

19 (0.8%) Refused

68 (2.8%) Other

2428 Households

**Household in the study clusters**

**Enumerated**

**Not Enumerated**

Households

9 m - <5 years children

5 - <15 years children

563

1238

539 (95.7%)

1163 (93.9%)

351

351

Individuals

Enumerated

Available

Randomly Selected

Enrolled & Analysed

312 (88.8%)

324 (92.3%)

**Enumerated:** Visited all households in the cluster and collect identification details (name, date of birth/age, gender) **Available:** Children are available for the next three day**s Randomly selected:** Automated selection of children from enumeration data using android application. **Enrolled and analysed:** Data collected after obtaining consent/assent

**Supplementary Figure 1-C: Flowchart describing the enrolment of participants in the post-SIA serosurveys by district**

**Palghar, Maharashtra**

**Post-SIA survey**

3297 Households

2832 (85.9%) households

465 (14.1%) Households

310 (9.4%) Locked

126 (3.8%) Refused

29 (0.9%) Other

**Household in the study clusters**

**Enumerated**

**Not Enumerated**

Households

9 m - <5 years children

5 - <15 years children

778

1885

724 (93.1%)

1667 (88.4%)

387

390

Individuals

Enumerated

Available

Randomly Selected

336 (86.8%)

319 (81.8%)

Enrolled & Analysed

**Enumerated:** Visited all households in the cluster and collect identification details (name, date of birth/age, gender) **Available:** Children are available for the next three day**s Randomly selected:** Automated selection of children from enumeration data using android application. **Enrolled and analysed:** Data collected after obtaining consent/assent

**Supplementary Figure 1-D: Flowchart describing the enrolment of participants in the post-SIA serosurveys by district**

**Kanpur Nagar, Uttar Pradesh**

**Post-SIA survey**

2907 Households

2528 (87%) households

379 (13%) Households

179 (6.2%) Locked

85 (2.9%) Refused

115 (3.9%) Other

**Household in the study clusters**

**Enumerated**

**Not Enumerated**

Households

9 m - <5 years children

5 - <15 years children

799

1935

683 (85.5%)

1581 (81.7%)

390

390

Individuals

Enumerated

Available

Randomly Selected

341 (87.4%)

350 (89.7%)

Enrolled & Analysed

**Enumerated:** Visited all households in the cluster and collect identification details (name, date of birth/age, gender) **Available:** Children are available for the next three day**s Randomly selected:** Automated selection of children from enumeration data using android application. **Enrolled and analysed:** Data collected after obtaining consent/assent

**Supplementary Figure 1-E: Flowchart describing the enrolment of participants in the post-SIA serosurveys by district**

**Thiruvananthapuram, Kerala**

**Post-SIA survey**

3671 Households

2832 (77.1%) households

839 (22.9%) Households

782 (21.3%) Locked

3 (0.08%) Refused

54 (1.5%) Other

**Household in the study clusters**

**Enumerated**

**Not Enumerated**

Households

9 m - <5 years children

5 - <15 years children

570

1237

538 (94.4%)

1123 (90.8%)

387

390

Individuals

Enumerated

Available

Randomly Selected

340 (87.8%)

338 (86.6%)

Enrolled & Analysed

**Enumerated:** Visited all households in the cluster and collect identification details (name, date of birth/age, gender) **Available:** Children are available for the next three day**s Randomly selected:** Automated selection of children from enumeration data using android application. **Enrolled and analysed:** Data collected after obtaining consent/assent
