# Supplementary figures and images for: Post-campaign coverage evaluation of a measles and rubella supplementary immunization activity in five districts in India, 2019–2020
Source: PLoS One. 2024 Mar 29;19(3):e0297385. doi: 10.1371/journal.pone.0297385 (PMC10980234; doi:10.1371/journal.pone.0297385)

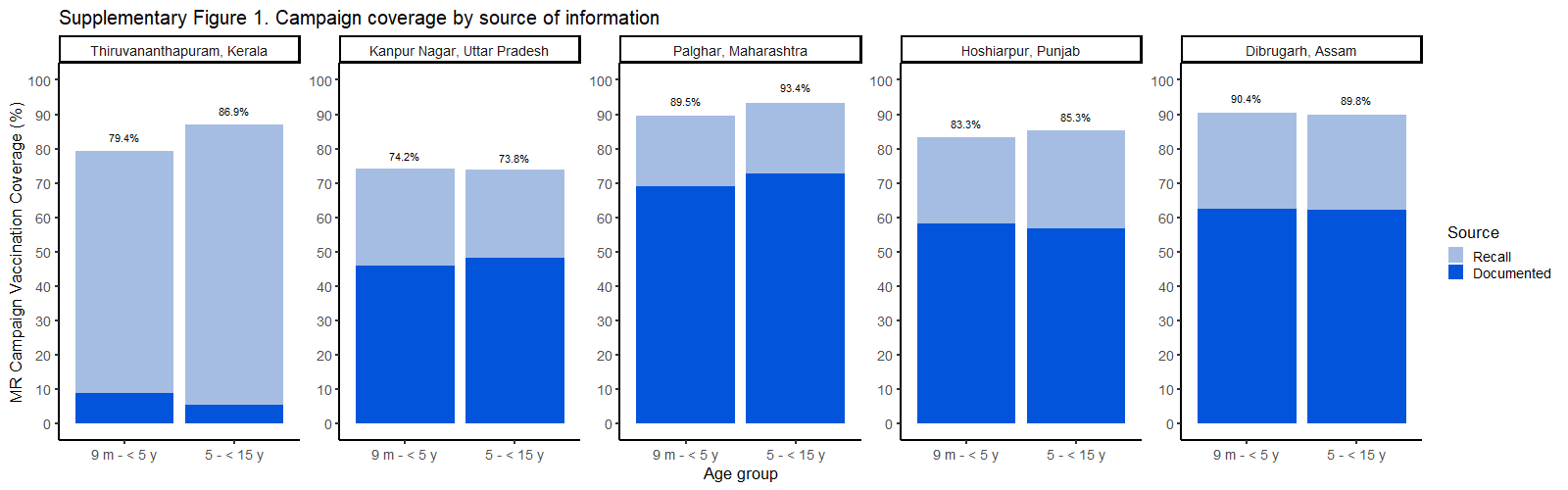

Supplement: S2 Fig — Survey weighted coverage estimates. (TIF) [file pone.0297385.s003.tif]

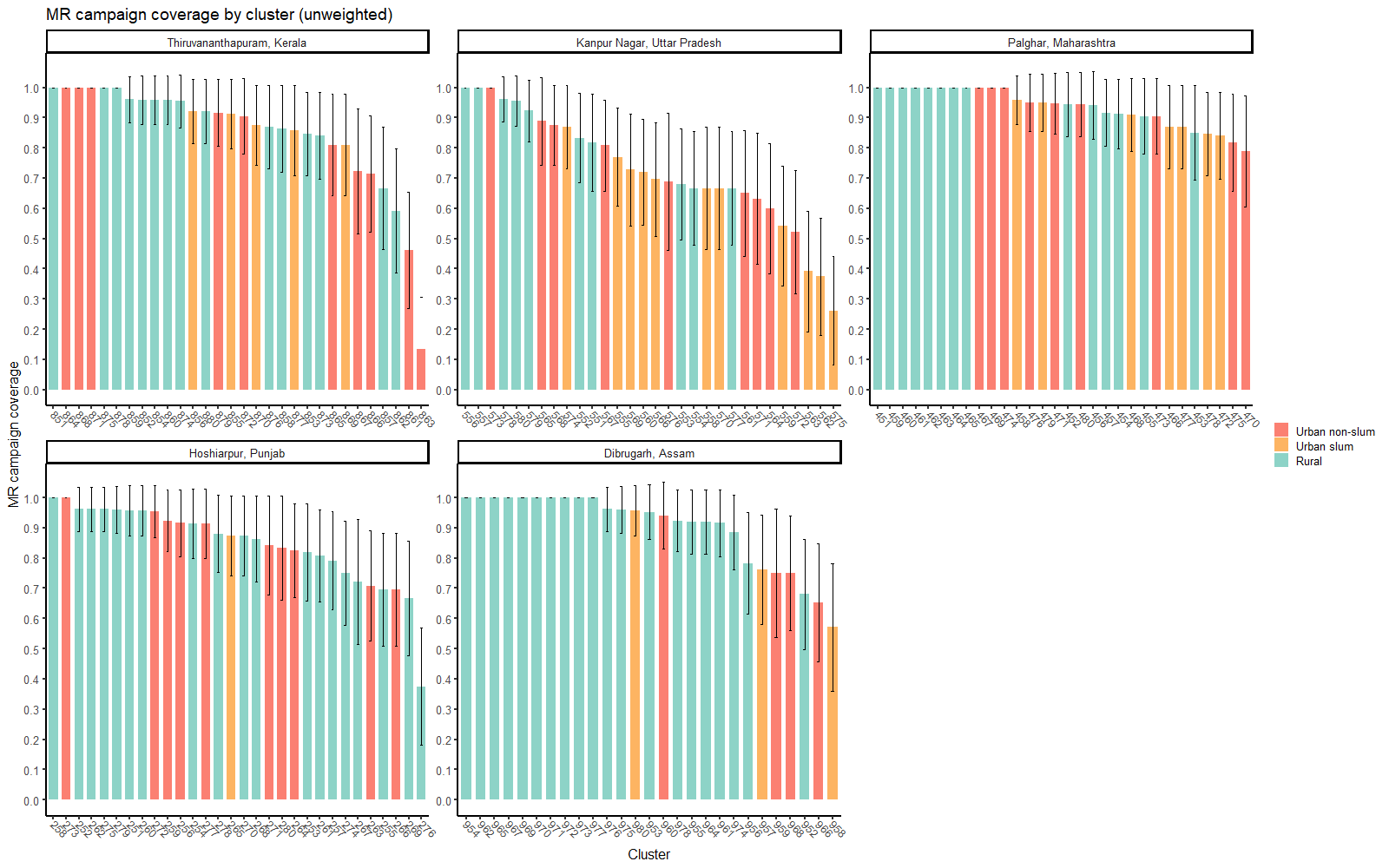

Supplement: S3 Fig — Unweighted coverage estimates. (TIF) [file pone.0297385.s004.tif]
