# Supplementary material for: Post-campaign coverage evaluation of a measles and rubella supplementary immunization activity in five districts in India, 2019–2020
Source: PLoS One. 2024 Mar 29;19(3):e0297385. doi: 10.1371/journal.pone.0297385 (PMC10980234; doi:10.1371/journal.pone.0297385)
Supplement: S1 Table — (DOCX) [file pone.0297385.s005.docx]

**Supplementary Table 1.** **Measles-rubella campaign vaccine coverage**

|  | **Thiruvananthapuram District, Kerala** | **Kanpur Nagar District, Uttar Pradesh** | **Palghar District, Maharashtra** | **Hoshiarpur District, Punjab** | **Dibrugarh District, Assam** |
| --- | --- | --- | --- | --- | --- |
|  | **% (95% CI)** | **% (95% CI)** | **% (95% CI)** | **% (95% CI)** | **% (95% CI)** |
| Campaign coverage^a^ |  |  |  |  |  |
| 9 months to < 5 years |  |  |  |  |  |
| Documented | 8.7 (5.5, 13.5) | 45.9 (34.0, 58.4) | 69.1 (60.2, 76.7) | 58.1 (48.0, 67.5) | 62.4 (50.1, 73.2) |
| Recall only | 70.7 (58.8, 80.3) | 28.2 (20.9, 36.9) | 20.4 (14.4, 28.2) | 25.2 (17.3, 35.3) | 28.1 (18.3, 40.5) |
| Card+recall | 79.4 (65.6, 88.7) | 74.2 (64.1, 82.2) | 89.5 (85.1, 92.7) | 83.3 (74.3, 89.6) | 90.4 (83.7, 94.6) |
| Design effect (card+recall) | 6.6 | 3.5 | 1.2 | 3.4 | 2.4 |
| 5 to < 15 years |  |  |  |  |  |
| Documented | 5.2 (2.7, 9.6) | 48.2 (37.4, 59.2) | 72.8 (65.0, 79.4) | 56.8 (47.9, 65.3) | 62.3 (49.6, 73.5) |
| Recall only | 81.7 (75.0, 87.0) | 25.6 (18.0, 35.0) | 20.6 (15.0, 27.6) | 28.4 (21.4, 36.6) | 27.5 (18.5, 38.8) |
| Card+recall | 86.9 (79.9, 91.7) | 73.8 (63.2, 82.2) | 93.4 (88.6, 96.3) | 85.3 (79.5, 89.6) | 89.8 (80.1, 95.1) |
| Design effect (card+recall) | 2.4 | 3.9 | 1.7 | 1.7 | 4.3 |
| Campaign coverage by number of prior doses received^b^ |  |  |  |  |  |
| ‘True’ zero-dose | 49.0 (15.5, 83.5) | 45.7 (21.8, 71.8) | 83.4 (47.2, 96.6) | 81.2 (41.2, 96.4) | 93.7 (73.1, 98.8) |
| One | 63.2 (40.7, 81.1) | 71.7 (56.2, 83.4) | 87.8 (75.0, 94.5) | 83.2 (64.4, 93.2) | 83.4 (70.0, 91.5) |
| Two | 85.6 (73.7, 92.6) | 79.4 (69.8, 86.5) | 90.4 (83.4, 94.6) | 88.0 (82.5, 91.9) | 91.8 (80.2, 96.9) |
| Unknown | 40.4 (11.2, 78.5) | 53.3 (21.2, 82.9) | 91.7 (29.9, 99.7) | 55.2 (29.5, 78.4) | 95.3 (84.5, 98.7) |

1. Survey weighted coverage estimates.
2. Coverage of routine immunization doses based on card plus recall. Restricted to children 9 months to below 5 years of age at time of the campaign (those with routine immunization data). ‘True’ zero-dose indicates vaccination status reported as not having received any doses (Kerala, N = 9; Uttar Pradesh, N = 24; Maharashtra, N = 20; Punjab, N = 11; Assam, N = 24) vs. unknown for children missing vaccination status (Kerala, N = 14; Uttar Pradesh, N = 13; Maharashtra, N = 10; Punjab, N = 33; Assam, N = 65).

Coverage significantly higher among older children compared to younger children in Thiruvananthapuram District, Kerala (p < 0.05). No other significant differences were observed.
