# Supplementary material for: Post-campaign coverage evaluation of a measles and rubella supplementary immunization activity in five districts in India, 2019–2020
Source: PLoS One. 2024 Mar 29;19(3):e0297385. doi: 10.1371/journal.pone.0297385 (PMC10980234; doi:10.1371/journal.pone.0297385)
Supplement: S2 Table — (DOCX) [file pone.0297385.s006.docx]

**Supplementary Table 2. Primary source of information about measles-rubella campaign**

|  | **Health worker**  **n (%)** | **Newspaper/TV/radio n (%)** | **Social media**  **n (%)** | **School or community member n (%)** | **Was not aware**  **n (%)** |
| --- | --- | --- | --- | --- | --- |
| **Thiruvananthapuram District, Kerala** | 220 (32.5) | 22 (3.2) | 21 (3.1) | 348 (51.3) | 48 (7.1) |
| **Kanpur Nagar District, Uttar Pradesh** | 248 (35.9) | 3 (0.4) | 0 (0.0) | 358 (51.8) | 75 (10.9) |
| **Palghar District, Maharashtra** | 321 (48.9) | 4 (0.6) | 4 (0.6) | 287 (43.8) | 26 (4.0) |
| **Hoshiarpur District, Punjab** | 203 (29.3) | 4 (0.6) | 8 (1.2) | 388 (55.9) | 81 (11.7) |
| **Dibrugarh District, Assam** | 398 (62.5) | 0 (0.0) | 0 (0.0) | 196 (30.8) | 15 (2.4) |

Row percentages. Reflects the first field on source of information. Additional options (“don’t remember” and “other”) were included in summary but excluded from this table (< 2%).
