# Supplementary material for: Post-campaign coverage evaluation of a measles and rubella supplementary immunization activity in five districts in India, 2019–2020
Source: PLoS One. 2024 Mar 29;19(3):e0297385. doi: 10.1371/journal.pone.0297385 (PMC10980234; doi:10.1371/journal.pone.0297385)
Supplement: S3 Table — (DOCX) [file pone.0297385.s007.docx]

**Supplementary Table 3. Campaign coverage by awareness of campaign**

|  | Received campaign dose | | | | | | | | | | | | | | | | | |
| --- | --- | --- | --- | --- | --- | --- | --- | --- | --- | --- | --- | --- | --- | --- | --- | --- | --- | --- |
| Aware of campaign | Thiruvananthapuram, Kerala | | | Kanpur Nagar, Uttar Pradesh | | | Palghar, Maharashtra | | | Hoshiarpur, Punjab | | | Dibrugarh, Assam | | | All Sites | | |
|  | N | Yes | No | N | Yes | No | N | Yes | No | N | Yes | No | N | Yes | No | N | Yes | No |
| Yes | 630 | 576 (91.4) | 54 (8.6) | 616 | 505 (82.0) | 111 (18.0) | 630 | 613 (97.3) | 17 (2.7) | 613 | 587 (95.8) | 26 (4.2) | 622 | 576 (92.6) | 46 (7.4) | 3111 | 2857 (91.8) | 254 (8.2) |
| No | 48 | 5 (10.4) | 43 (89.6) | 75 | 2 (2.7) | 73 (97.3) | 26 | 1 (3.9) | 25 (96.2) | 81 | 1 (1.2) | 80 (98.8) | 15 | 1 (6.7) | 14 (93.3) | 245 | 10 (4.1) | 235 (95.9) |

Percentages reflect campaign receipt stratified by awareness of the campaign (row percentages).
