# Supplementary material for: Post-campaign coverage evaluation of a measles and rubella supplementary immunization activity in five districts in India, 2019–2020
Source: PLoS One. 2024 Mar 29;19(3):e0297385. doi: 10.1371/journal.pone.0297385 (PMC10980234; doi:10.1371/journal.pone.0297385)
Supplement: S4 Table — (DOCX) [file pone.0297385.s008.docx]

**Supplementary Table 4. Factors associated with receipt of the campaign dose, district-specific univariable logistic regression models**

|  | **Thiruvananthapuram, Kerala** | | | **Kanpur Nagar, Uttar Pradesh** | | | **Palghar, Maharashtra** | | | **Hoshiarpur, Punjab** | | | **Dibrugarh, Assam** | | |
| --- | --- | --- | --- | --- | --- | --- | --- | --- | --- | --- | --- | --- | --- | --- | --- |
| **Characteristic** | **OR**^1^ | **95% CI**^1^ | **p-value** | **OR**^1^ | **95% CI**^1^ | **p-value** | **OR**^1^ | **95% CI**^1^ | **p-value** | **OR**^1^ | **95% CI**^1^ | **p-value** | **OR**^1^ | **95% CI**^1^ | **p-value** |
| **Sex** |  |  | 0.43 |  |  | 0.44 |  |  | 0.56 |  |  | 0.50 |  |  | **0.10** |
| *Female* | — | — |  | — | — |  | — | — |  | — | — |  | — | — |  |
| *Male* | 1.19 | 0.76, 1.87 |  | 1.10 | 0.85, 1.44 |  | 0.77 | 0.30, 1.94 |  | 1.15 | 0.76, 1.73 |  | 0.66 | 0.39, 1.11 |  |
| **Maternal education** |  |  | **0.068** |  |  | **0.014** |  |  | **0.11** |  |  | **0.032** |  |  | 0.42 |
| *Graduate and above* | — | — |  | — | — |  | — | — |  | — | — |  | — | — |  |
| *Middle to higher secondary* | 1.61 | 0.54, 4.83 |  | 0.81 | 0.37, 1.76 |  | 3.68 | 0.78, 17.3 |  | 0.98 | 0.47, 2.05 |  | 0.72 | 0.28, 1.84 |  |
| *Primary* | 0.31 | 0.07, 1.39 |  | 0.83 | 0.28, 2.41 |  | 1.59 | 0.25, 10.0 |  | 1.65 | 0.42, 6.54 |  | 1.07 | 0.17, 6.73 |  |
| *Illiterate* | 1.13 | 0.24, 5.38 |  | 0.40 | 0.16, 0.99 |  | 1.51 | 0.35, 6.48 |  | 0.36 | 0.10, 1.30 |  | 0.43 | 0.05, 3.97 |  |
| **Head of household occupation** |  |  | **0.042** |  |  | **0.026** |  |  | 0.27 |  |  | 0.44 |  |  | **<0.001** |
| *Professional, technician, clerks* | — | — |  | — | — |  | — | — |  | — | — |  | — | — |  |
| *Service & sales workers, agriculture, craft trade* | 1.90 | 0.44, 8.21 |  | 0.90 | 0.43, 1.89 |  | 0.81 | 0.24, 2.74 |  | 0.51 | 0.14, 1.88 |  | 1.80 | 0.85, 3.81 |  |
| *Unemployed* | 0.71 | 0.15, 3.49 |  | 0.36 | 0.16, 0.79 |  | 2.89 | 0.39, 21.6 |  | 0.73 | 0.14, 3.73 |  | 0.23 | 0.02, 2.33 |  |
| **Setting** |  |  | 0.36 |  |  | **0.004** |  |  | **0.085** |  |  | **0.078** |  |  | **<0.001** |
| *Urban non-slum* | — | — |  | — | — |  | — | — |  | — | — |  | — | — |  |
| *Urban, slum* | 2.09 | 0.65, 6.73 |  | 0.44 | 0.19, 1.01 |  | 0.65 | 0.36, 1.17 |  | 1.29 | 0.82, 2.02 |  | 2.50 | 0.83, 7.50 |  |
| *Rural* | 2.36 | 0.63, 8.78 |  | 1.84 | 0.78, 4.36 |  | 1.59 | 0.64, 3.96 |  | 0.80 | 0.41, 1.57 |  | 9.64 | 4.71, 19.7 |  |
| **Religion** |  |  | **0.16** |  |  | **0.087** |  |  | **0.009** |  |  | **<0.001** |  |  | 0.84 |
| *Hindu* | — | — |  | — | — |  | — | — |  | — | — |  | — | — |  |
| *Muslim or Christian* | 0.60 | 0.28, 1.26 |  | 0.43 | 0.15, 1.18 |  | 0.19 | 0.06, 0.67 |  | 0.11 | 0.04, 0.31 |  | 0.84 | 0.14, 5.00 |  |
| *Sikhs / Buddhist / Jain* |  |  |  |  |  |  | 0.45 | 0.13, 1.56 |  | 0.83 | 0.46, 1.51 |  | — | — |  |
| **Caste** |  |  | 0.94 |  |  | **0.23** |  |  | 0.27 |  |  | 0.76 |  |  | 0.92 |
| *General / Other Backward Class* | — | — |  | — | — |  | — | — |  | — | — |  | — | — |  |
| *Scheduled Caste / Tribe* | 0.96 | 0.32, 2.87 |  | 0.66 | 0.33, 1.33 |  | 1.61 | 0.66, 3.93 |  | 1.08 | 0.65, 1.79 |  | 0.95 | 0.29, 3.13 |  |
| **Type of schooling** |  |  | **<0.001** |  |  | **0.014** |  |  | **<0.001** |  |  | **<0.001** |  |  | **<0.001** |
| *Public* | — | — |  | — | — |  | — | — |  | — | — |  | — | — |  |
| *Private* | 0.71 | 0.25, 2.00 |  | 0.90 | 0.49, 1.65 |  | 2.50 | 0.47, 13.4 |  | 0.89 | 0.36, 2.24 |  | 0.47 | 0.20, 1.10 |  |
| *Does not attend school* | 797,914 | 208,795, 3,049,238 |  | 0.16 | 0.05, 0.55 |  | 0.31 | 0.11, 0.87 |  | 0.02 | 0.00, 0.20 |  | 0.17 | 0.05, 0.63 |  |
| *Too young* | 0.27 | 0.12, 0.61 |  | 0.89 | 0.46, 1.73 |  | 0.53 | 0.24, 1.18 |  | 0.17 | 0.06, 0.48 |  | 0.33 | 0.12, 0.92 |  |
| **Age at campaign (years)** | 1.05 | 0.98, 1.12 | **0.14** | 0.98 | 0.92, 1.03 | 0.40 | 1.01 | 0.91, 1.13 | 0.79 | 0.99 | 0.90, 1.08 | 0.74 | 0.97 | 0.88, 1.07 | 0.51 |

Results from district-specific survey weighted logistic regression models. Bold p-values indicate p < 0.25. Due to small sample size in the Sikhs / Buddhist / Jain category in Dibrugarh District, Assam (N=1) one child was excluded from the regression analysis.
