# Supplementary material for: Post-campaign coverage evaluation of a measles and rubella supplementary immunization activity in five districts in India, 2019–2020
Source: PLoS One. 2024 Mar 29;19(3):e0297385. doi: 10.1371/journal.pone.0297385 (PMC10980234; doi:10.1371/journal.pone.0297385)
Supplement: S5 Table — (DOCX) [file pone.0297385.s009.docx]

**Supplementary Table 5. Factors associated with receipt of the campaign dose, district-specific multivariable logistic regression models**

|  | **Thiruvananthapuram, Kerala** | | | **Kanpur Nagar, Uttar Pradesh** | | | **Palghar, Maharashtra** | | | **Hoshiarpur, Punjab** | | | **Dibrugarh, Assam** | | |
| --- | --- | --- | --- | --- | --- | --- | --- | --- | --- | --- | --- | --- | --- | --- | --- |
| **Characteristic** | **OR**^1^ | **95% CI**^1^ | **p-value** | **OR**^1^ | **95% CI**^1^ | **p-value** | **OR**^1^ | **95% CI**^1^ | **p-value** | **OR**^1^ | **95% CI**^1^ | **p-value** | **OR**^1^ | **95% CI**^1^ | **p-value** |
| **Sex** |  |  | -- |  |  | -- |  |  | -- |  |  | -- |  |  | **0.027** |
| *Female* | -- | -- |  | -- | -- |  | -- | -- |  | -- | -- |  | -- | -- |  |
| *Male* | -- | -- |  | -- | -- |  | -- | -- |  | -- | -- |  | 0.60 | 0.37, 0.96 |  |
| **Maternal education** |  |  | **0.012** |  |  | 0.49 |  |  | **0.048** |  |  | **0.028** |  |  | -- |
| *Graduate and above* | -- | -- |  | -- | -- |  | -- | -- |  | -- | -- |  | -- | -- |  |
| *Middle to higher secondary* | 1.00 | 0.60, 1.66 |  | 0.77 | 0.40, 1.47 |  | 2.09 | 0.48, 9.06 |  | 0.90 | 0.40, 2.05 |  | -- | -- |  |
| *Primary* | 0.15 | 0.04, 0.52 |  | 0.92 | 0.38, 2.22 |  | 0.45 | 0.08, 2.61 |  | 2.97 | 0.62, 14.2 |  | -- | -- |  |
| *Illiterate* | 0.33 | 0.02, 4.35 |  | 0.58 | 0.25, 1.30 |  | 0.28 | 0.05, 1.60 |  | 0.46 | 0.13, 1.57 |  | -- | -- |  |
| **Head of household occupation** |  |  | **0.030** |  |  | 0.24 |  |  | -- |  |  | -- |  |  | **0.009** |
| *Professional, technician, clerks* | -- | -- |  | -- | -- |  | -- | -- |  | -- | -- |  | -- | -- |  |
| *Service & sales workers, agriculture, craft trade* | 2.21 | 0.78, 6.24 |  | 1.20 | 0.62, 2.32 |  | -- | -- |  | -- | -- |  | 1.00 | 0.45, 2.25 |  |
| *Unemployed* | 0.73 | 0.25, 2.09 |  | 0.59 | 0.27, 1.30 |  | -- | -- |  | -- | -- |  | 0.17 | 0.03, 0.97 |  |
| **Setting** |  |  | -- |  |  | **0.002** |  |  | **<0.001** |  |  | 0.16 |  |  | **<0.001** |
| *Urban non-slum* | -- | -- |  | -- | -- |  | -- | -- |  | -- | -- |  | -- | -- |  |
| *Urban, slum* | -- | -- |  | 0.67 | 0.32, 1.44 |  | 1.06 | 0.54, 2.10 |  | 2.65 | 0.91, 7.72 |  | 1.86 | 0.63, 5.51 |  |
| *Rural* | -- | -- |  | 2.11 | 1.00, 4.48 |  | 5.55 | 1.90, 16.2 |  | 0.98 | 0.56, 1.74 |  | 10.2 | 4.34, 23.9 |  |
| **Religion** |  |  | 0.14 |  |  | 0.079 |  |  | **<0.001** |  |  | **0.001** |  |  | -- |
| *Hindu* | -- | -- |  | -- | -- |  | -- | -- |  | -- | -- |  | -- | -- |  |
| *Muslim or Christian* | 0.68 | 0.40, 1.15 |  | 0.52 | 0.24, 1.10 |  | 0.13 | 0.03, 0.60 |  | 0.12 | 0.04, 0.39 |  | -- | -- |  |
| *Sikhs / Buddhist / Jain* | -- | -- | -- | -- | -- | -- | 0.24 | 0.12, 0.50 |  | 0.74 | 0.41, 1.34 |  | -- | -- |  |
| **Caste** |  |  | -- |  |  | 0.10 |  |  | -- |  |  | -- |  |  | -- |
| *General / Other Backward Class* | -- | -- |  | — | — |  | -- | -- |  | -- | -- |  | -- | -- |  |
| *Scheduled Caste / Tribe* | -- | -- |  | 0.66 | 0.39, 1.11 |  | -- | -- |  | -- | -- |  | -- | -- |  |
| **Type of schooling** |  |  | **<0.001** |  |  | 0.086 |  |  | **<0.001** |  |  | **<0.001** |  |  | **0.007** |
| *Public* | -- | -- |  | -- | -- |  | -- | -- |  | -- | -- |  | -- | -- |  |
| *Private* | 0.70 | 0.36, 1.37 |  | 0.73 | 0.42, 1.27 |  | 3.70 | 0.98, 14.0 |  | 0.75 | 0.35, 1.62 |  | 1.06 | 0.52, 2.15 |  |
| *Does not attend school* | 1,016,691 | 278,741, 3,708,312 |  | 0.26 | 0.09, 0.75 |  | 0.31 | 0.07, 1.36 |  | 0.02 | 0.00, 0.15 |  | 0.17 | 0.05, 0.55 |  |
| *Too young* | 0.27 | 0.13, 0.58 |  | 0.76 | 0.38, 1.50 |  | 0.46 | 0.18, 1.14 |  | 0.15 | 0.04, 0.63 |  | 0.31 | 0.09, 1.04 |  |
| **Age at campaign (years)** | 1.03 | 0.94, 1.12 | 0.49 | -- | -- | -- | -- | -- | -- | -- | -- | -- | -- | -- | -- |
| ***Goodness of fit for final models*** | | | | | | | | | | | | | | | |
| **AIC value** | 569.9 | | | 739.0 | | | 301.0 | | | 529.9 | | | 346.7 | | |
| **Logistic regression**  **c-stat (or area**  **under the ROC**  **curve)** | 0.71 | | | 0.69 | | | 0.79 | | | 0.69 | | | 0.76 | | |

Results from district-specific survey weighted multivariable logistic regression models including all variables with p < 0.25 from univariable models. Bold p-values indicate p < 0.05. Due to small sample size in the Sikhs / Buddhist / Jain category in Dibrugarh District, Assam (N=1) one child was excluded from the regression analysis.
