# Supplementary material for: Post-campaign coverage evaluation of a measles and rubella supplementary immunization activity in five districts in India, 2019–2020
Source: PLoS One. 2024 Mar 29;19(3):e0297385. doi: 10.1371/journal.pone.0297385 (PMC10980234; doi:10.1371/journal.pone.0297385)
Supplement: S6 Table — (DOCX) [file pone.0297385.s010.docx]

**Supplementary Table 6. Campaign coverage by sex and age group**

| Age group | Sex | Thiruvananthapuram, Kerala | | Kanpur Nagar,  Uttar Pradesh | | Palghar, Maharashtra | | Hoshiarpur, Punjab | | | | Dibrugarh, Assam | |  |
| --- | --- | --- | --- | --- | --- | --- | --- | --- | --- | --- | --- | --- | --- | --- |
|  |  | % (95% CI) | p-value | % (95% CI) | p-value | % (95% CI) | p-value | | % (95% CI) | p-value | % (95% CI) | | p-value | |
| 9 months - < 5 years | Female | 75.9  (59.0, 87.3) | 0.053 | 69.3  (56.6, 79.7) | 0.051 | 91.1  (82.9, 95.6) | 0.47 | | 82.2  (71.7, 89.4) | 0.56 | 92.5  (85.4, 96.3) | | 0.12 | |
|  | Male | 82.6  (70.3, 90.5) |  | 79.0 (68.9, 86.4) |  | 87.8  (81.3, 92.3) |  |  | 84.4  (74.6, 90.9) |  | 88.9  (81.0, 93.7) | |  |  |
| 5 - < 15 years | Female | 86.8  (77.2, 92.7) | 0.94 | 74.6  (63.2, 83.4) | 0.62 | 94.1 (85.2, 97.8) | 0.76 | | 84.4  (77.3, 89.6) | 0.59 | 91.8  (82.9, 96.2) | | 0.20 | |
|  | Male | 87.1  (79.5, 92.1) |  | 73.2  (62.3, 81.8) |  | 92.8 (84.4, 96.8) |  |  | 86.1  (79.5, 90.8) |  | 88.0  (75.4, 94.6) | |  |  |

Survey weighted coverage estimates. P-values from survey weighted district-specific univariable logistic regression models.
