# Supplementary material for: Post-campaign coverage evaluation of a measles and rubella supplementary immunization activity in five districts in India, 2019–2020
Source: PLoS One. 2024 Mar 29;19(3):e0297385. doi: 10.1371/journal.pone.0297385 (PMC10980234; doi:10.1371/journal.pone.0297385)
Supplement: S7 Table — (DOCX) [file pone.0297385.s011.docx]

**Supplementary Table 7. Location of measles-rubella campaign receipt, by child’s age at the time of the campaign**

| **Site** | **N** | **School n (%)** | **Government:**  **Health Facility site n (%)** | **Government: Outreach session n (%)** | **Other n (%)** |  |
| --- | --- | --- | --- | --- | --- | --- |
| **Children 9 m - < 3 years** | | | | | | |
| Thiruvananthapuram District, Kerala | 145 | 19 (13.1) | 88 (60.7) | 32 (22.1) | 6 (4.1) |  |
| Kanpur Nagar District, Uttar Pradesh | 120 | 20 (16.7) | 37 (30.8) | 61 (50.8) | 2 (1.7) |  |
| Palghar District, Maharashtra | 178 | 16 (9.0) | 59 (33.1) | 102 (57.3) | 1 (0.6) |  |
| Hoshiarpur District, Punjab | 129 | 61 (47.3) | 27 (20.9) | 41 (31.8) | 0 (0.0) |  |
| Dibrugarh District, Assam | 130 | 87 (66.9) | 10 (7.7) | 30 (23.1) | 3 (2.3) |  |
| **Children 3 - < 15 years** | | | | | | |
| Thiruvananthapuram District, Kerala | 436 | 333 (76.4) | 74 (17) | 25 (5.7) | 4 (0.9) |  |
| Kanpur Nagar District, Uttar Pradesh | 387 | 290 (74.9) | 13 (3.4) | 80 (20.7) | 4 (1.0) |  |
| Palghar District, Maharashtra | 436 | 318 (72.9) | 45 (10.3) | 73 (16.7) | 0 (0.0) |  |
| Hoshiarpur District, Punjab | 459 | 369 (80.4) | 38 (8.3) | 52 (11.3) | 0 (0.0) |  |
| Dibrugarh District, Assam | 447 | 376 (84.1) | 20 (4.5) | 49 (11) | 2 (0.4) |  |

Government health facility was defined as primary health center, community health center, subdistrict hospital, or district hospital. Government outreach session was defined as Anganwadi center and Panchayat Bhawan, or a mobile site. Some children 9 months - < 3 years may be attending school per their parent or caregiver.
