# Supplementary material for: Post-campaign coverage evaluation of a measles and rubella supplementary immunization activity in five districts in India, 2019–2020
Source: PLoS One. 2024 Mar 29;19(3):e0297385. doi: 10.1371/journal.pone.0297385 (PMC10980234; doi:10.1371/journal.pone.0297385)
Supplement: S8 Table — (DOCX) [file pone.0297385.s012.docx]

**Supplementary Table 8. Location of measles-rubella campaign receipt, by school attendance**

|  | **Where child attends school** | | | | | | | | | | | |
| --- | --- | --- | --- | --- | --- | --- | --- | --- | --- | --- | --- | --- |
|  | **Public n (%)** | | | **Private n (%)** | | | **Too young n (%)** | | | **Does not attend n (%)** | | |
| **Where child received MR campaign** | **School** | **Govt:**  **Health Facility** | **Govt: Outreach** | **School** | **Govt:**  **Health Facility** | **Govt: Outreach** | **School** | **Govt:**  **Health Facility** | **Govt: Outreach** | **School** | **Govt:**  **Health Facility** | **Govt: Outreach** |
| Thiruvananthapuram District, Kerala | 217 (62.9) | 95 (27.5) | 31 (9.0) | 131 (71.2) | 32 (17.4) | 16 (8.7) | 4 (8.7) | 31 (67.4) | 9 (19.6) | 0 (0.0) | 4 (66.7) | 1 (16.7) |
| Kanpur Nagar District, Uttar Pradesh | 82 (75.2) | 2 (1.8) | 24 (22) | 211 (74.8) | 13 (4.6) | 55 (19.5) | 14 (14.0) | 34 (34.0) | 50 (50.0) | 3 (18.8) | 1 (6.3) | 12 (75.0) |
| Palghar District, Maharashtra | 161 (88.5) | 11 (6.0) | 10 (5.5) | 152 (97.4) | 1 (0.6) | 3 (1.9) | 11 (6.5) | 57 (33.9) | 99 (58.9) | 10 (9.3) | 35 (32.4) | 63 (58.3) |
| Hoshiarpur District, Punjab | 129 (83.8) | 9 (5.8) | 16 (10.4) | 295 (72.3) | 49 (12.0) | 64 (15.7) | 6 (25.0) | 6 (25.0) | 12 (50.0) | 0 (0.0) | 1 (50.0) | 1 (50.0) |
| Dibrugarh District, Assam | 345 (81.9) | 15 (3.6) | 59 (14) | 61 (84.7) | 1 (1.4) | 9 (12.5) | 45 (67.2) | 9 (13.4) | 11 (16.4) | 12 (70.6) | 5 (29.4) | 0 (0.0) |

In some contexts, a school may have served as an outreach vaccination site for the campaign. The school where the child received the MR campaign dose may not be the same as where the child goes to school.

Significant difference in location of receipt comparing children attending public vs. private schools in Thiruvananthapuram District, Kerala, Palghar District, Maharashtra, Hoshiarpur District, Punjab, with private school students more likely to receive the campaign vaccine dose in school in Thiruvananthapuram and Palghar districts, and converse in Hoshiarpur district.

Due to confusion during the conduct of the survey between ‘does not attend’ and ‘too young’ options, 3 years of age was used to distinguish ‘too young’ from ‘does not attend’ for analysis purposes. Children less than 3 years old at the time of the campaign who were marked as ‘does not attend’ were considered ‘too young’ for the analysis and children 3 years or older at the time of the campaign who were marked as ‘too young’ were considered ‘does not attend’ for analysis purposes.
