# Supplementary material for: Post-campaign coverage evaluation of a measles and rubella supplementary immunization activity in five districts in India, 2019–2020
Source: PLoS One. 2024 Mar 29;19(3):e0297385. doi: 10.1371/journal.pone.0297385 (PMC10980234; doi:10.1371/journal.pone.0297385)
Supplement: S9 Table — (PDF) [file pone.0297385.s013.pdf]

Supplementary Table 9. Reasons for not receiving the campaign vaccine, children aged 9 months to less than 15 years, restricted to children aware of the campaign

Site	N	Lack of knowledge of campaign	Lack of faith in vaccines or vaccine campaigns	Lack of time or other issue	Private provider or provider advised against campaign	Fear of pain and other side effects or fear of vaccines	Heard from social media vaccine may harm child	Heard from news or community member vaccine may harm	School opted out	Child sick	Did not live in state during MR campaign	Dont remember	Other
Thiruvananthapuram, Kerala	54	16.7	5.6	1.9	11.1	9.3	5.6	1.9	3.7	11.1	7.4	16.7	9.3
Kanpur Nagar, Uttar Pradesh	110	7.3	7.3	10.9	2.7	24.5	0.9	8.2		15.5	21.8		0.9
Palghar, Maharashtra	13			7.7	15.4	7.7		7.7		7.7	30.8	15.4	7.7
Hoshiarpur, Punjab	26	19.2	23.1				23.1		11.5	11.5	7.7		3.8
Dibrugarh, Assam	46	28.3		4.3		4.3				26.1	4.3	17.4	15.2
All Sites	249	14.1	6.8	6.4	4.4	14.1	4	4.4	2	15.7	14.5	7.6	6

Other includes service delivery issue and heard vaccine may cause harm from religious leader.

Table is restricted to children whose caregivers reported being aware of the campaign. When asked why the child had not received the MR campaign dose in a subsequent question some caregivers indicated lack of knowledge about the campaign as the reason. This may have included situations where the caregiver was aware of the campaign but was not aware of the logistics such as when or where to have their child vaccinated.
